# GPR18 Deletion in Mice Inversely Affects Vasoactive Signaling and Passive Biomechanical Properties of the Thoracic Aorta and Femoral Artery

**DOI:** 10.3390/ijms27020841

**Published:** 2026-01-14

**Authors:** Sofie De Moudt, Ameziane Herzine, Marc-Damien Lourenco Rodrigues, Maud Collin, Magnus Bäck, Frances T. Yen, Nathalie Mercier

**Affiliations:** 1 Université de Lorraine, Inserm, DCAC, F-54000 Nancy, Franceameziane.herzine@univ-orleans.fr (A.H.); marc-damien.lourenco-rodrigues@inserm.fr (M.-D.L.R.); nathalie.mercier@univ-lorraine.fr (N.M.); 2 Université de Lorraine, Animalerie du Campus Biologie Santé, F-54000 Nancy, France; maud.collin@univ-lorraine.fr

**Keywords:** GPR18, myography, vasoconstriction

## Abstract

The G protein-coupled receptor GPR18, engaged by pro-resolving and cannabinoid-related lipid ligands, plays a vascular bed-specific protective role in endothelial function. The aim of the present study was to establish the vasoreactivity and passive biomechanical properties of the thoracic aorta and femoral artery of adult GPR18 knockout compared with wildtype mice, using ex vivo myography, arterial morphology, and immunohistochemistry. The results revealed heightened receptor-independent contractility, loss of prostanoid-dependent contractile responses, altered vascular smooth muscle cell (VSMC) calcium handling, and an attenuated stress–tension relationship in the thoracic aorta of GPR18 knockout mice. This phenotype was almost entirely reversed in the femoral artery, with attenuated receptor-independent contractility, unchanged VSMC calcium handling, and a heightened stress–tension relationship in GPR18 knockout mice. These vascular bed-specific differences highlight the need to consider tissue context in the development of GPR18-based vasculoprotective therapies for cardiovascular disease.

## 1. Introduction

Unresolved chronic inflammation has been identified as an important emerging risk factor in cardiovascular disease (CVD). This stems from a failure to resolve the inflammatory process due to an imbalance between pro-inflammatory mediators and specialized pro-resolving mediators (SPMs). The latter can transduce active cell signals as ligands for G protein-coupled receptors (GPRs), initiating a series of cellular responses, which promote the attenuation of inflammatory activity to re-establish tissue homeostasis [1,2]. Therefore, pro-resolution treatment for both acute and chronic CVD could represent an interesting strategy to reduce CVD risk without interfering with appropriate immune responses that are crucial for protection against harmful stimuli.

SPMs are derived from long-chain polyunsaturated fatty acids (PUFAs). One SPM of interest is resolvin (Rv)D2, which is derived from the n-3 PUFA, docosahexaenoic acid (DHA), and interacts with its specific receptor GPR18. Since administration of RvD2 was shown to prevent murine atherosclerotic plaque development in a GPR18-dependent manner [3,4], this pathway is of particular interest in CVD prevention. A number of studies have demonstrated the resolving properties of the GPR18/RvD2 axis in inflammatory cells such as macrophages and neutrophils in cardiovascular inflammation [2,4,5,6]. Recent evidence suggests that GPR18/RvD2 signaling can also directly affect vascular-specific cell types, i.e., endothelial cells (ECs) and vascular smooth muscle cells (VSMCs) [7]. RvD2-mediated stimulation of GPR18 led to increased vasorelaxant substances including nitric oxide (NO) and prostacyclin [8]. Our recent results showed that GPR18 deletion induced endothelial dysfunction related to NO and eNOS expression in femoral arteries altering the endothelial-dependent vasodilatation associated with hypertension in aged mice [9]. Together, this suggests a direct role of the GPR18/RvD2 axis in the regulation of vasodilation through both NO synthase (NOS) and cyclooxygenase (COX)-dependent mechanisms. Similar results were obtained for other GPR18 agonists (endogenous and synthetic), including N-arachidonylglycine (NAGly) [10,11,12,13], abnormal cannabidiol (Abn-CBD) [11,14], PSB-MZ-1415 [11], PSB-MZ-1440 [11], and PSB-KD-107 [15].

These observations led us to investigate a potential direct role of GPR18 in vasoactive cell signaling by measuring the vasoconstrictive reactivity and passive biomechanical properties of young adult GPR18-deficient mice in elastic and muscular-type arteries.

## 2. Results

### 2.1. GPR18 Expression in the Arterial Wall of the Thoracic Aorta and Femoral Artery

**Figure 1:**
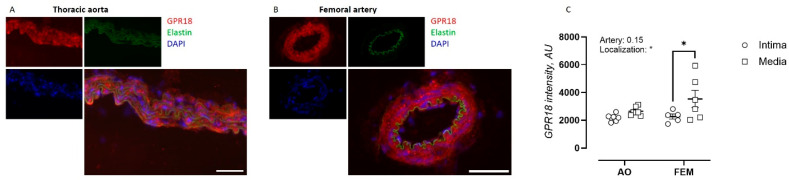
GPR18 expression in the arterial wall of the thoracic aorta (AO) and femoral artery (FEM). Immunofluorescent staining for GPR18 (red), autofluorescent elastin signal (green), and DAPI nuclear staining (blue) for the thoracic aorta (**A**) and femoral artery (**B**) of GPR18 WT mice and their quantification within the medial and intimal layer (**C**). Scale bars represent 50 µm. Statistical analysis using two-way ANOVA. * *p* < 0.05.

To investigate the arterial site-specific nature of endothelial dysfunction in GPR18 KO mice, the distribution of GPR18 levels within the arterial wall was determined by immunofluorescent staining on sections of the thoracic aorta and femoral artery from WT C57Bl/6 mice. GPR18 was found to be expressed in both the intimal and medial layers of the thoracic aorta (Figure 1A) and femoral artery (Figure 1B). Interestingly, relative GPR18 staining was significantly higher in the medial layer compared with the intimal layer of the femoral artery.

### 2.2. GPR18 Deletion Inversely Affects Contractility and VSMC Calcium Handling of the Thoracic Aorta and Femoral Artery

**Figure 2:**
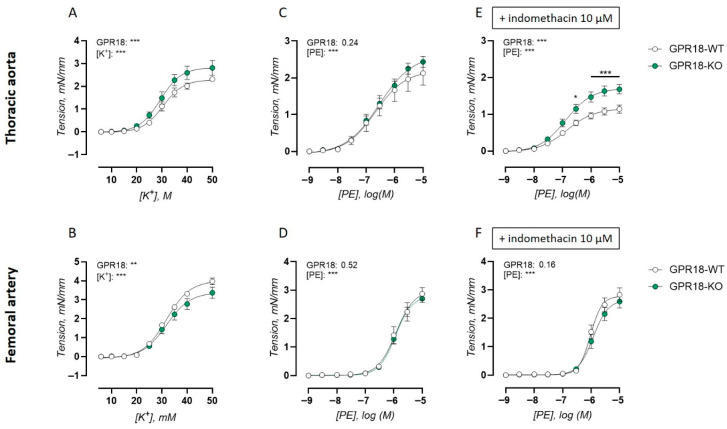
Contractile responses in the isolated thoracic aorta and femoral artery of GPR18 KO mice. Receptor-independent contractions were measured as a concentration–response curve in response to extracellular K^+^ in arterial rings of the thoracic aorta (WT, *K*^+^7; KO, *n* = 7) (**A**,**C**,**E**) and femoral artery (WT, *n* = 7; KO, *n* = 8) (**B**,**D**,**F**). α1-adrenoreceptor-dependent contractions were measured as a concentration–response curve in response to PE in arterial rings of the thoracic aorta (WT, *n* = 8; KO, *n* = 8) (**C**,**E**) and femoral artery (WT, *n* = 7; KO, *n* = 10) (**D**,**F**) in the absence (**C**,**D**) and presence (**E**,**F**) of COX inhibitor indomethacin (10 µM). Statistical analysis using two-way ANOVA. * *p* < 0.05; ** *p* < 0.01; *** *p* < 0.001.

To investigate the contractile function of the isolated thoracic aorta and femoral artery, receptor-independent contractions were measured in response to increasing extracellular K^+^ concentrations. K^+^-induced concentration–response curves are presented in Figure 2A,B and non-linear fit variables for sensitivity (EC_50_) and maximal effect (Emax) are shown in Table 1. This revealed heightened contractions in the thoracic aorta of the GPR18 KO mice (Figure 2A), whereas contractile responses were reduced in the femoral artery of the GPR18 KO mice (Figure 2B).

Next, α1-adrenoreceptor-dependent contractions were measured in response to concentration–response stimulation with PE. This measurement was performed in the absence and presence of non-selective COX inhibitor indomethacin, to assess the contribution of vasoactive prostanoid signaling. PE-induced concentration–response curves are presented in Figure 2C–F and non-linear fit variables for sensitivity (EC_50_) and maximal effect (Emax) are shown in Table 2.

Unlike K^+^-induced contractile responses, no difference in PE-induced concentration–contraction curves was found between genotypes in baseline conditions (Figure 2C). However, when COX was inhibited using indomethacin, the concentration–contraction curves were significantly elevated in the GPR18 KO mice (Figure 2E). In general, COX inhibition by indomethacin reduced PE-induced contractile responses in the thoracic aorta, represented by a significantly decreased Emax value (Table 2). This effect was less pronounced in the GPR18 KO mice, demonstrating a loss of prostanoid-dependent vasocontraction.

In the femoral artery, no differences between genotypes in PE-induced concentration–contraction curves were observed in baseline conditions (Figure 2D) or in the presence of indomethacin (Figure 2F). Similarly, COX inhibition did not significantly alter either the maximal effect or sensitivity of PE-induced contractions of the femoral artery (Table 2).

To investigate VSMC contractility in more depth, calcium influx pathways, baseline VSMC tone, and contractile responses upon intracellular calcium release were investigated. During these measurements, 300 µM L-NAME was always present to inhibit possible confounding effects of NO production on vasoactive VSMC signaling.

**Figure 3:**
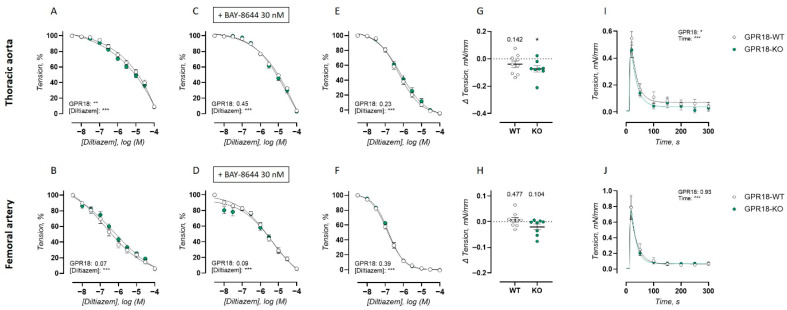
VSMC calcium handling in the isolated thoracic aorta and femoral artery of GPR18 KO mice. Arterial relaxations in response to VGCC inhibition by diltiazem were measured in 10 µM PE precontracted arterial rings in the absence (**A**,**B**) and presence (**C**,**D**) of VGCC agonist BAY-K8644 (30 nM), or after precontraction using 50 mM K^+^ (**E**,**F**) for the thoracic aorta (WT, *n* = 8; KO, *n* = 8) (**A**,**C**,**E**) and femoral artery (WT, *n* = 8; KO, *n* = 9) (**B**,**D**,**F**). Baseline VSMC tonus was determined as the decrease in tension when replacing normal Krebs–Ringer solution with a solution lacking calcium in arterial rings of the thoracic aorta (**G**) and femoral artery (**H**). Tracings of SR-mediated transient contractions after 10 µM PE stimulation in the absence of extracellular calcium are shown for arterial rings of the thoracic aorta (WT, *n* = 8; KO, *n* = 8) (**I**) and femoral artery (WT, *n* = 8; KO, *n* = 9) (**J**). During these measurements, 300 µM L-NAME was always present to inhibit possible confounding effects of NO production on vasoactive VSMC signaling. Statistical analysis using two-way ANOVA (**A**–**F**,**I**,**J**) or one-sample *t*-test against a hypothetical value of 0 (**G**,**H**). * *p* < 0.05; ** *p* < 0.01; *** *p* < 0.001.

As the main source of calcium influx during α1-adrenergic contraction, VGCC contribution was investigated by concentration–response inhibition using diltiazem in PE-precontracted arterial rings. This measurement was performed in the absence and presence of VGCC agonist BAY-K8644, to maximally activate VGCC. Finally, diltiazem-induced concentration–relaxation curves were also measured in 50 mM K^+^-precontracted arterial rings, since this contraction is almost exclusively dependent on VGCC-dependent calcium entry [16,17]. Diltiazem-induced concentration–relaxation curves are presented in Figure 3A–F.

In the thoracic aorta, concentration–response inhibition by diltiazem induced a larger relaxation in the GPR18 KO mice compared to the WT controls (Figure 3A), demonstrating a higher contribution of VGCC to PE-induced contractions. This observed difference was lost after maximal activation of the VGCC with 30 nM BAY-K8644 (Figure 3C) or when arterial rings were precontracted by 50 mM K^+^. Unlike the thoracic aorta, no significant differences between genotypes were observed in the diltiazem concentration–response curves of the femoral artery, independent of the precontraction (Figure 3B,D,F).

Next, basal VSMC tone was investigated by replacing the Krebs–Ringer solution with a solution lacking calcium. In the thoracic aorta of the GPR18 KO mice, this introduced a significant drop in tension, which was absent in the WT controls (Figure 3G), indicating a significant basal VSMC tone. Unlike the thoracic aorta, no significant effect of calcium removal was observed in the femoral artery of the GPR18 KO or WT mice (Figure 3H).

Finally, contractions due to the release of sarcoplasmic reticulum (SR) calcium stores were investigated by 10 µM PE stimulation of the arterial rings in a calcium-free environment. This revealed reduced SR-mediated contractions in the thoracic aorta of the GPR18 KO mice compared to the WT control (Figure 3I). Unlike the thoracic aorta, no significant difference between genotypes was observed in the SR-mediated contractions of the femoral artery (Figure 3J).

### 2.3. GPR18 KO Mice Display Inverse Changes in Passive Biomechanical Properties in the Thoracic Aorta and Femoral Artery

**Figure 4:**
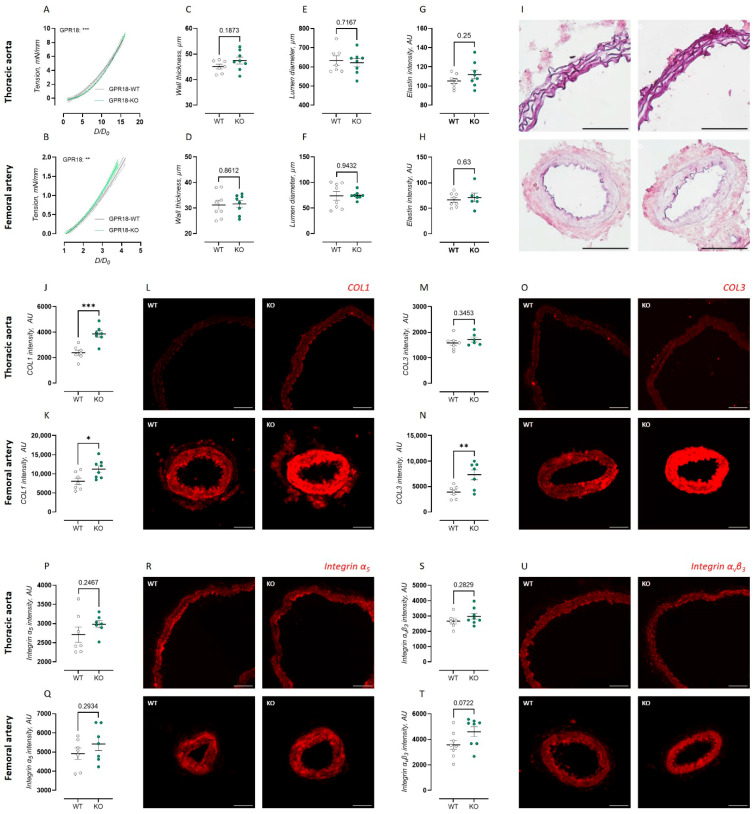
Biomechanical properties and wall composition of the thoracic aorta and femoral artery of GPR18 KO mice. Passive arterial wall distensibility was assessed as the stress–tension relationship, with D/D_0_ representing relative diameter change, in Krebs–Ringer solution of the thoracic aorta (WT, *n* = 9; KO, *n* = 9) (**A**) and femoral artery (WT, *n* = 8; KO, *n* = 10) (**B**). Vessel dimensions, i.e., wall thickness (**C**,**D**) and lumen diameter (**E**,**F**), were measured by delineating the internal and external elastic laminae using the autofluorescent signal of elastin in the thoracic aorta (**C**,**E**) and femoral artery (**D**,**F**). Quantification of orcein staining intensity (**G**,**H**) and immunofluorescent intensity for collagen type 1 (**J**,**K**), collagen type 3 (**M**,**N**), integrin α5 (**P**,**Q**), and integrin αvβ3 (**S**,**T**) in the thoracic aorta (**G**,**J**,**M**,**P**,**S**) and femoral artery (**H**,**K**,**N**,**Q**,**T**). Representative images of orcein staining (**I**) and immunofluorescent staining for collagen type 1 (**L**), collagen type 3 (**O**), integrin α5 (**R**), and integrin αvβ3 (**U**) in the thoracic aorta (upper panel) and femoral artery (lower panel) of GPR18 WT and KO mice. Scale bars represent 100 µm (thoracic aorta immunofluorescence) or 50 µm (femoral artery, thoracic aorta orcein). Data are expressed as quadric regression curve ± 95% CI (**A**,**B**) or mean ± SEM (**C**–**T**). Statistical analysis using sum-of-squares F-test (**A**,**B**) or Student’s *t*-test (**C**–**T**). * *p* < 0.05; ** *p* < 0.01; *** *p* < 0.001.

To investigate the passive arterial biomechanical properties of isolated arterial rings of the thoracic aorta and femoral artery, progressive static stretch was applied to measure the passive tension development. Stress–tension curves are presented in Figure 4A,B. The thoracic aorta of the GPR18 KO mice displayed a significantly lower stress–tension relationship compared to the WT controls (Figure 4A), demonstrating increased arterial compliance. On the other hand, the femoral artery of the GPR18 KO mice showed a decreased stress–tension relationship compared to the WT controls (Figure 4B), demonstrating increased arterial stiffness.

To investigate a possible mechanism behind these changes in arterial biomechanical behavior in the GPR18 KO mice, the arterial wall dimensions, ECM composition, and cell adhesion markers were investigated. In both arterial sites, no significant differences in vessel dimensions were observed between genotypes (Figure 4C–F). Furthermore, the orcein staining intensity to measure the elastin content was not significantly different between genotypes (Figure 4G–I). Immunofluorescent staining for collagen type 1 revealed a significantly increased mean signal intensity in the thoracic aorta of the GPR18 KO mice (Figure 4J), whereas the collagen type 3 intensity was not significantly different between genotypes in this artery (Figure 4M). At the level of the femoral artery, the immunofluorescent staining intensity for both collagen type 1 (Figure 4K) and type 3 (Figure 4N) was significantly increased in the GPR18 KO mice compared to the GPR18 WT controls. No significant differences between genotypes were observed in immunofluorescent staining intensity for the cell adhesion markers integrin-α5 and integrin-αvβ3 in the studied vascular beds (Figure 4P–U).

## 3. Discussion

The current study extends the recent establishment of endothelial dysfunction in GPR18-deficient mice [9] by showing the direct effects of GPR18 deletion on vasoconstriction, arterial stiffness, and ECM remodeling. These vascular bed-specific differences may guide future efforts to develop GPR18-targeted approaches to preserve vascular function in cardiovascular disease.

GPR18 was expressed in both the intimal and medial layers of the arterial wall. This is in line with previous in vitro findings, where GPR18 expression has also been detected in both ECs [18,19] and VSMCs [20]. Although higher GPR18 protein levels have previously been described in mouse large conductance arteries compared to small resistance arteries [7], no differences in the overall GPR18 protein levels were observed in the present study between the murine thoracic aorta and femoral artery. However, the present study revealed an uneven distribution of GPR18 protein levels within the arterial wall, with higher levels in the medial than the intimal layer, which was most pronounced in the femoral artery.

Reduced vasoconstriction by COX inhibition in the thoracic aorta supports an important modulation of vasoreactivity through contractile prostanoids in this arterial segment. Interestingly, GPR18 deletion significantly reduced the contribution of contractile prostanoid signaling to PE-induced contractions, thereby compensating for the heightened receptor-independent contractility of this artery. In contrast, relaxant prostanoids prevailed in the femoral artery, in which indomethacin reduced acetylcholine-induced vasorelaxation [9]. In this study, these observations were extended by showing a lack of effects of COX inhibition on contractile responses in the femoral artery, highlighting an important production of relaxant prostanoids in this artery. It is apparent that the shift towards COX-dependent contractile modulation in the femoral artery of the GPR18 KO mice was thus largely driven by VSMCs. Another indication that GPR18 and prostanoid vasoactive pathways may be interconnected is that GPR18 agonism by RvD2 can prevent TXA2-induced contractions specifically, without affecting the α1 adrenoreceptor-induced contractility of the rat thoracic aorta [21].

Systemic GPR18 deletion heightened the receptor-independent contractility of the thoracic aorta. This was accompanied by a multitude of changes in VSMC calcium handling, including increased activation of VGCC, an active basal VSMC tonus, and reduced SR-mediated contractions. All the abovementioned findings may be explained by increased cytosolic calcium concentrations. Although this was not directly measured in the present study, the fact that the active basal VSMC tonus could be reversed by the removal of extracellular calcium is a clear indication that cytosolic calcium could be increased in the thoracic aorta VSMC of GPR18 KO mice. Interestingly, this phenotype was completely absent in the femoral artery, where attenuated contractility was shown in the GPR18 KO mice but VSMC calcium handling was otherwise preserved. This pronounced difference in the effect of GPR18 deletion on contractile signaling between large elastic and muscular-type arteries would suggest that targeting hypercontractility in arterial diseases through GPR18 signaling would be most effective in cardiovascular diseases that primarily affect large elastic arteries (e.g., systolic hypertension, arterial stiffening). Indeed, similar results were found in a murine model of hypertension, where GPR18 agonism by RvD2 could prevent hypercontractility in the thoracic aorta only, whereas no beneficial effects were observed in the small mesenteric arteries [22]. This confirms that the vascular bed-specific regulation of VSMC contractility by GPR18 may result in regional differences in the therapeutic effect of GPR18 agonism.

So far, very limited information is available regarding the role of GPR18 in arterial stiffness regulation. Although RvD2 improved vascular dysfunction in AngII-treated mice, either in the absence [22] or presence [7] of dyslipidemia, no protective effect of GPR18 agonism through systemic RvD2 delivery was observed on the arterial stiffening of the small mesenteric arteries. However, since arterial stiffening is a disease that primarily occurs in the elastic arteries, more focused studies are needed to address this question. In the present study, we describe significant effects of GPR18 deletion on the passive biomechanical properties of both the elastic and muscular arteries, through the measurement of their stress–tension relationship. Interestingly, the effect of GPR18 deletion on arterial stiffness is inversed between both arterial sites, with increased distensibility in the thoracic aorta and reduced distensibility in the femoral artery. This could therefore lead to a heightened stiffness gradient across the arterial tree in GPR18 KO mice. Calcium handling in VSMCs also affects the extracellular matrix, and the current study revealed increased medial collagen type 1 and 3 levels in the femoral artery of the GPR18 KO mice, providing a mechanism for the observed increase in arterial stiffness. In the context of atherosclerosis, increased collagen depositions have been described upon systemic RvD2 administration [23]. Therefore, collagen levels may increase both after GPR18 deletion and GPR18 agonism. In the thoracic aorta, only collagen type 1 levels were significantly increased in the GPR18 KO mice. This is in contradiction with the functional observation of a reduced stress–tension relationship. Measurements of vessel dimensions, elastin content, and cell adhesion markers could not provide a possible mechanism for this observation. Considering the uneven distribution of GPR18 immunohistochemical staining intensity between the intimal and medial layers, nondestructive testing of different arterial layers during ex vivo myography analysis could potentially offer future insight into the role of GPR18 in the regulation of biomechanical arterial behavior. Although the systemic consequences of these biomechanical changes have not been investigated, they may include altered left ventriculo-arterial coupling (leading to left ventricular remodeling), changes in central blood pressure, or changes in blood flow at the level of the microcirculation. Confounding reports are available on the effect of GPR18 agonism on blood pressure regulation in normotensive animals. Systemic delivery of RvD2 was shown not to affect the blood pressure of healthy mice [22]. However, we recently reported GPR18 deletion induced hypertension in older, but not in young mice [9]. Hypotensive response was observed upon delivery of other GPR18 agonists, such as NAGly [12], Abn-CBD [14], and PSB-KD-107 [15]. Of interest, some of these effects seem to be GPR18-independent [12] or (at least partially) related to central nervous system blood pressure regulation [24,25].

The underlying signaling pathway of GPR18 could be cell type-dependent, agonist-dependent, or may even display plasticity depending on (patho)physiological conditions. Moreover, multiple G proteins have been found to couple with GPR18, including Gi/o [26] and Gq [26,27]. This could potentially explain the disparate findings observed in the present study between elastic and muscular arteries, which, however, remain to be established. The further insight into this pathway provided by the present study could be of important clinical significance in the prevention and treatment of cardiovascular disease.

## 4. Materials and Methods

### 4.1. Laboratory Animals

Heterozygous GPR18+/− (MGI:107859; NCBI ID: 110168) (official name, C57BL/6J-Gpr18em1cyagen) mice were purchased from Taconic Biosciences (Model No. TF1875), and bred to obtain wildtype GPR18+/+ (WT) and GPR18−/− knockout (KO) mice. All mice were bred and housed in the certified animal facility Animalerie du Campus Biologie Santé (ACBS) of the University of Lorraine (approval number C-5454730) under standard conditions (22 °C, 12 h/12 h day/night cycle, 70% humidity) and with free access to water and standard chow. A total of 54 mice were used in this study, including 28 GPR18 knockout mice (male, *n* = 8; female, *n* = 20) and 26 littermate GPR18 wildtype control mice (male, *n* = 9; female, *n* = 17). Mice were euthanized by cervical dislocation under isoflurane anesthesia (4% in oxygen) at a mean age of 3.58 ± 0.05 months for tissue harvesting. Randomization and blinding were not applicable to the study design and selected outcome measures.

### 4.2. Ex Vivo Isometric Vasoreactivity

After an overdose of anesthesia, the thoracic aorta and left femoral artery from mice were quickly removed and stripped of adherent tissue. Vessel segments of 2 mm length were mounted in a four-channel pin (aorta) or wire (femoral artery) myograph (DMT, Hinnerup, Denmark) and immersed in Krebs–Ringer solution (composition [mM]: NaCl 118; KCl 4.7; CaCl_2_ 2.5; KH_2_PO_4_ 1.2; MgSO_4_ 1.2; NaHCO_3_ 25.0; CaEDTA 0.025; glucose 11.1). The solution was aerated with a 95% O_2_/5%CO_2_ gas mixture in a 37 °C water bath, and maintained at pH 7.4. After equilibration, the vessel segments were gradually stretched to a stress above 13.3 kPa (100 mmHg), using incremental steps of 100 µm (thoracic aorta) or 20 µm (femoral artery), to determine the stress–tension relation by calculation of the relative diameter change (D/D_0_) and vessel tension as previously described [28]. Afterwards, pretension was set to 13.3 kPa stress (normalization factor = 0.9) [29] and force transducers were reset to zero in order to measure active tension upon the addition of vasoactive agents. Contractile tension was measured using LabChart 7 software and reported in mN/mm [30]. Where possible, arteries of GPR18 KO and GPR18 WT mice were studied in parallel.

Receptor-independent contractions were generated using Krebs–Ringer solutions with stepwise increases in potassium (5.9–50 mM K^+^), obtained by iso-osmotic replacement of NaCl with KCl. Receptor-dependent contractions were induced by α1-adrenergic agonist phenylephrine (PE, 3 nM to 10 µM) in the absence and presence of indomethacin (10 µM), and in the absence and presence of L-NAME (300 µM).

Voltage-gated calcium channel (VGCC) contribution to vasocontraction was studied by cumulative concentration–response stimulation with VGCC antagonist diltiazem (10 nM to 100 mM) in arterial rings precontracted with 10 µM PE in the presence and absence of VGCC agonist BAY-K8644 (30 nM), or in 50 mM K^+^-precontracted arterial rings. Intracellular calcium handling was studied in a calcium-free Krebs–Ringer solution to avoid extracellular calcium influx, by induction of transient contractions using 10 µM PE stimulation. These contractions result from the inositol triphosphate (IP3)-dependent release of contractile calcium from the sarcoplasmic reticulum (SR), as previously described [16,17,31,32,33].

All measurements were performed after steady-state conditions were reached. For inhibitory concentration–response curves, all data were normalized against the value of tension during precontraction. Concentration–response curves were fitted using a non-linear four-parameter equation where appropriate to assess the maximal effect (Emax) and the half-maximal effective concentration (EC_50_).

### 4.3. Immunohistochemistry

Cryosections of OCT-embedded thoracic aorta and femoral artery samples of 8 GPR18 KO mice (male, *n* = 4; female, *n* = 4) and 8 GPR18 WT mice (male, *n* = 4; female, *n* = 4) were fixed using 4% paraformaldehyde for immunofluorescent assessment of GPR18 (Abcam, Cambridge, UK, ab76258), collagen type-1 (Abcam, ab138492), collagen type-3 (Thermofisher, Villebon Courtaboeux, France, PA5-99160), integrin α5 (Chemicon, Temecula, CA, USA, AB1928), and integrin αvβ3 (Bioss, Woburn, MA, USA, bs-1310R). Localization in the intimal or medial layer was assessed using the autofluorescent signal of medial elastin. All images were acquired using a Nikon Eclipse Ci-S fluorescence microscope (Champigny-sur-Marne, France) and analyzed using ImageJ software (version 1.53c).

### 4.4. Histological Orcein Staining

A standard orcein staining was performed on 10 µm cryosections of OCT-embedded thoracic aorta and femoral artery samples of 8 GPR18 KO mice (male, *n* = 4; female, *n* = 4) and 8 GPR18 WT mice (male, *n* = 4; female, *n* = 4). All images were acquired using a Nikon Eclipse Ti2 light microscope (Champigny-sur-Marne, France) and analyzed using ImageJ software.

### 4.5. Statistical Analysis

Data are expressed as mean ± SEM unless otherwise specified; the number of biological replicates is indicated by n. GraphPad Prism software (version 9) was used for statistical analysis; statistical significance was considered as *p* < 0.05. Normality of data was verified using the Shapiro–Wilk test and (non-)parametric testing was applied when appropriate. For ANOVA statistical analyses, post hoc testing was performed using Šídák’s multiple comparisons test.

## 5. Conclusions

To conclude, GPR18 is unevenly distributed in the arterial wall and GPR18 deletion inversely affects the vasoactive signaling and passive biomechanical properties of the thoracic aorta and femoral artery in healthy adult mice. These results demonstrate that GPR18 is necessary to maintain normal vascular physiology. Although the mechanism behind vascular bed-specific contractile modulations through GPR18 remains unclear, this may have important implications for the further development of pro-resolution treatments for cardiovascular disease through GPR18 agonism.

## Figures and Tables

**Table 1:** Non-linear fit variables of K^+^-induced contractions in the thoracic aorta and femoral artery of GPR18 KO mice.

	WT	KO	*p*-Value
*n*			
Thoracic aorta	7	7	
Femoral artery	7	8	
EC_50_, log(M)			
Thoracic aorta	31.53 ± 1.37	29.76 ± 0.78	0.28
Femoral artery	32.15 ± 0.42	32.69 ± 0.98	0.55
E_max_, mN/mm			
Thoracic aorta	2.44 ± 0.13	2.91 ± 0.37	0.25
Femoral artery	4.2 ± 0.22	3.56 ± 0.28	0.10

Abbreviations: EC_50_, half-maximal excitatory concentration; Emax, maximal effect; KO, knockout; WT, wildtype. Data are represented as mean ± SEM. Statistical analysis using a Student’s ***t***-test.

**Table 2:** Non-linear fit variables of PE-induced contractions in the thoracic aorta and femoral artery of GPR18 KO mice.

	WT-Baseline	WT-Indo	KO-Baseline	KO-Indo	Overall *p*-Value		
					GPR18	Indo	Interaction
*n*							
Thoracic aorta	8	7	8	7			
Femoral artery	7	7	10	10			
EC_50_, log(M)							
Thoracic aorta	−6.6 ± 0.11	−6.83 ± 0.07	−6.54 ± 0.13	−6.87 ± 0.07 (^#^)	0.93	*	0.62
Femoral artery	−5.84 ± 0.17	−5.98 ± 0.06	−5.94 ± 0.06	−5.87 ± 0.11	0.93	0.74	0.34
E_max_, %							
Thoracic aorta	2.2 ± 0.32	1.16 ± 0.12 (^##^)	2.62 ± 0.19	1.71 ± 0.13 (^##^)	*	***	0.76
Femoral artery	3.3 ± 0.41	2.81 ± 0.22	2.77 ± 0.13	2.67 ± 0.18	0.16	0.22	0.40

Abbreviations: EC_50_, half-maximal excitatory concentration; E_max_, maximal effect; GPR18, G protein-coupled receptor 18; indo, indomethacin; KO, knockout; PE, phenylephrine; WT, wildtype. Data are represented as mean ± SEM. Statistical analysis using two-way ANOVA with Šídák’s multiple comparisons test for GPR18 (*) and/or indomethacin (^#^) as applicable. */^#^ *p* < 0.05; ^##^ *p* < 0.01; *** *p* < 0.001.

## Data Availability

The original contributions presented in this study are included in the article. Further inquiries can be directed to the corresponding authors.

## Abbreviations

The following abbreviations are used in this manuscript:

Abn-CBD	Abnormal cannabidiol
Ach	Acetylcholine
COX	Cyclooxygenase
CVD	Cardiovascular disease
DHA	Docosahexaenoic acid
EC_50_,	Half-maximal excitatory concentration
EC_max_	Maximal effect
ECM	Extracellular matrix
GPR	G protein-coupled receptor
Indo	Indomethacin
KO	Knockout
L-NAME	L-NG-Nitroarginine Methyl Ester
NAGlyc	N-arachidonylglycine
NO	Nitric oxide
NOS	Nitric oxide synthase
PE	Phenylepinephrine
PUFA	Polyunsaturated fatty acids
RvD2	Resolvin D2
SPM	Specialized pro-resolving mediators
VGCC	Voltage-gated calcium channel
VSMC	Vascular smooth muscle cells
WT	Wildtype
